# A new strategy for tuberculosis control in North Korea

**DOI:** 10.4178/epih/e2015053

**Published:** 2015-11-22

**Authors:** Young-jeon Shin, Moran Ki, Nackmoon Sung

**Affiliations:** 1 Department of Preventive Medicine, Hanyang University College of Medicine, Seoul, Korea; 2 Department of Cancer Control and Policy, Graduate School of Cancer Science and Policy, National Cancer Center, Goyang, Korea; 3 Clinical Research Center, Masan National Tuberculosis Hospital, Changwon, Korea

**Keywords:** Tuberculosis, Multi-drug resistant, Democratic People’s Republic of Korea, International cooperation

## Abstract

Tuberculosis is one of the most prevalent diseases in North Korea. Despite some positive accomplishments by current aid projects, it is still necessary to investigate the existing aid system. The following are necessary for improvement: sustaining a high degree of expertise, cooperation among various related parties including the international community, mediation to induce this cooperation, a more active role of the South Korean government, and encouragement of North Korea to more actively participate. Achieving these will help solve the issues of current tuberculosis aid projects in North Korea and lead to more successful outcomes.

## INTRODUCTION

Tuberculosis is one of the most prevalent diseases in Democratic People’s Republic of Korea (hereafter North Korea). In particular, tuberculosis receives targeted control efforts under the command of Il-Sung Kim. A management and treatment delivery system also independently contributes to the control of tuberculosis, along with hepatitis, in North Korea. In general, it has been known that the North Korean government had effectively controlled tuberculosis until the end of the 1970s.

Tuberculosis shows especially high prevalence and incidence in regions with poverty and famine. Economic and international political difficulties in North Korea at the end of the 1980s were followed by a great famine at the end of the 1990s, which resulted in the failure of tuberculosis control. In addition, tuberculosis requires long term, vigilant treatment, involving plenty of rest and nutrition. Without professional diagnoses and continuous monitoring, medications, and treatment by specialists for a long period of time, tuberculosis cannot be eradicated, and inadequate treatment often leads to resistant tuberculosis. Nevertheless, since the 1990s, it has been difficult for North Korea to find the resources that are required for effective diagnosis, treatment, and follow-up. For effective reconstruction of the North Korean tuberculosis control system, in this study we aimed to investigate the problems of the existing system and to find out strategic methods for improvement.

## CURRENT STATE OF TUBERCULOSIS AID PROJECTS IN NORTH KOREA

The tuberculosis aid projects in North Korea have been an important part of inter-Korean exchange and cooperation, especially when it comes to healthcare projects. The demand for and relatively low cost of treatment drugs in North Korea partly facilitated this exchange and cooperation. In addition, some inter-Korean cooperation organizations, such as “Eugene Bell,” which targets tuberculosis control in one of its projects, have made support for North Korea possible. Moreover, North Korea applied directly observed treatment, short course (DOTS) to the entire region of North Korea since 1997, and officially announced that it was highly successful. From 2010, a large financial support from the Global Fund allowed for the implementation of a comprehensive tuberculosis project for diagnosis, treatment, and control in the entire region of North Korea, and a new project (new strategic plan, 2015-2018) has recently been scheduled (Table 1).

Despite these large-scale projects, there is no solid evidence that tuberculosis in North Korea is under control. Rather, there are signs that many patients are unresponsive to tuberculosis treatments [1].

## MAJOR PROBLEMS OF TUBERCULOSIS CONTROL IN NORTH KOREA

The most serious problem of tuberculosis control in North Korea is the difficulty in securement of reliable tuberculosis-related data. Many questions have been raised regarding the reliability of pre-existing official data. Most of all, there is no regular nationwide investigation or surveillance.

For the diagnosis area of tuberculosis, North Korea is unable to conduct diagnosis through appropriate smear tests and radiography due to a shortage of basic equipment and medical professionals to check results of diagnosis. Thus, it is difficult for North Korea to reliably identify the nationwide incidence of tuberculosis and to systematically control tuberculosis patients. In particular, it has been known that North Korea has difficulty in the proper control of resistant tuberculosis patients due to extreme weaknesses in diagnostic abilities.

Regarding treatment, there is no confirmed evidence that the number of tuberculosis patients has significantly decreased despite the recent large expansion of aid projects. Currently, the biggest problem in treatment is the smear-negative patients. While there is a policy to control smear-positive patients continuously, there is no special control for smear-negative patients [2]. Since smear-negative patients are not quarantined in nursing facilities for a long time due to the shortages of food and residential resources even after diagnosis, these patients have a high chance of becoming the sources of infection to families or local communities. In addition, although a considerable number of multi-drug resistant tuberculosis (MDR-TB) patients exist, only some of them are under treatment, the validity of which has not yet been confirmed. Furthermore, the occurrence of extensively drug resistant tuberculosis (XDR-TB) patients was also reported recently [1].

On the other hand, since there is no effective mediation of roles and information sharing among the North Korean government, international organizations, and private organizations that participate in the tuberculosis aid projects in North Korea, the effect of the project has decreased. Particular problems in each organization are as follows.

### Tuberculosis project by international organizations including the Global Fund and the World Health Organization

#### Uncertain performance compared to the amount of input

The tuberculosis aid project in North Korea—a large scale project costing as much as 4,8021,294 US dollars—was executed from 2010 and received high praise in self-evaluation. However, it is difficult to conclude that the tuberculosis issue in North Korea and its control method have been significantly improved based on the North Korea tuberculosis-related indices reported both officially and unofficially.

Most of all, a wide-scale tuberculosis investigation, which can serve as a basis for planning, execution, and evaluation of projects, has not been completed. As such, we must rely on the inspection report by the World Health Organization (WHO) on the tuberculosis situation in North Korea (Report of the Joint Monitoring Mission, 2014), which suggests that there has been a partial improvement in the tuberculosis report rate [3]. However, this is still insufficient for evaluating the tuberculosis situation in North Korea and the effect of the tuberculosis control project. Although the ongoing baseline inspection is to be completed by the end of 2015 or later, it is unlikely to yield enough information to identify the performance of the project up to date. In addition, because the “New Strategic Plan” (new strategic plan 2015-2018) has been developed without national tuberculosis investigation results, it will most likely suffer from intrinsic limitations.

#### Problems in project promotion: “harmonization failure”

Although it was desirable to promote a large scale tuberculosis aid project in North Korea on the basis of the Global Fund, it was promoted by a so-called “forcing-in method,” without sufficient coordination with the existing organizations that have already established their own tuberculosis aid projects. This has caused the existing organizations to give up or change their projects suddenly, potentially wasting their members’ passions and capacities. Thereafter, although these pre-existing organizations sporadically supported anti-tuberculosis drugs either on small or large scales, these efforts were not integrated with large-scale projects operated by international organizations such as the WHO.

The issue is that these sporadic, unintegrated tuberculosis aid projects are highly likely to cause problems, including MDR-TB. Despite the continuous claims on this issue, the “harmonization failure” still seems unlikely to improve.

### Tuberculosis aid projects in North Korea by non-governmental organizations and the South Korean government

#### Unprofessionalism issue of supporting organizations

Tuberculosis aid projects, unlike other projects, require (1) a high degree of expertise, (2) field-oriented operation, (3) continuous careful monitoring and feedback, and (4) organic cooperation with administrative ability by the government. Without meeting these conditions, tuberculosis aid projects can result in problems such as MDR-TB and XDR-TB. If the control over MDR-TB fails, both South and North Koreas may face disastrous situations. It has already been reported that there has been a considerable number of MDR-TB patients in North Korea. Although the North Korean government should be primarily criticized for this, the international organizations and the South Korean domestic private organizations that had provided tuberculosis aids are also held responsible. Most of all, these situations indicate that there have been problems in the aid methods for tuberculosis in North Korea both in the past and at present. Furthermore, under the current situation, without improvement in primary diagnosis, treatment, or control of tuberculosis, some private organizations have begun using secondary drugs to treat MDR-TB. Information about the appropriateness of secondary drugs had not been made fully available by specialists. This may lead to increased incidence of XDR-TB in the future.

#### Lack of cooperation between supporting organizations

For the tuberculosis aid projects in North Korea, private organizations are rarely linked with the large-scale projects by the Global Fund/WHO and hardly share their functions. In addition, there is limited information exchange or cooperation between the private organizations, though they continue to raise funds and ship out anti-tuberculosis drugs using their own political powers.

#### Lack of the role of government as a mediator between organizations

At present, the South Korean government is unable to actively participate and coordinate projects operated by the international organizations and private organizations due to the shortage of medical staff and expertise on tuberculosis.

## CONCLUSION

### Political subjects and suggestions: medicine or poison?

While tuberculosis aid projects in North Korea are in need, these good-will efforts can become “poison,” rather than “medicine,” unless organized properly. Therefore, it is necessary to rapidly improve tuberculosis treatment problems in North Korea as follows.

### Rapid investigation on tuberculosis situations in North Korea and its regularization

For a more effective tuberculosis aid project, a nationwide investigation of the tuberculosis situation that has been delayed needs to be conducted as soon as possible, and future tuberculosis projects should be promoted based on the results of regular investigations on tuberculosis.

### Primary prevention and treatment-focused tuberculosis control project

Recently, the focus of tuberculosis aid in North Korea has shifted to the support for treatment of MDR-TB, which is a serious problem. A continuous nationwide tuberculosis investigation system should be implemented as soon as possible, and priority should be given to the goals of proper prevention and early detection and treatment, keeping in mind that “a stitch in time saves nine.”

### Prudent approach to the multi-drug resistant tuberculosis problem

It is highly risky to operate a MDR-TB treatment system using secondary anti-tuberculosis drugs before confirming the evidence of effective primary prevention and treatment of tuberculosis. Furthermore, the control of MDR-TB requires a high degree of expertise and close cooperation between related organizations, as additional resistance may cause severe adverse drug reactions. Thus, treatment should be implemented with full agreement between the Ministry of Health in North Korea and international organizations rather than the private organizations. Proper conditions for treatment should also be confirmed.

### Construction of ‘new governance’ for tuberculosis control in North Korea

In addition to appreciation of North Korean tuberculosis-related efforts and passion in the past and the present, it is necessary to objectively evaluate the control methods with the question, “Is the past and current way the best?” For now, the most important goal for effective control of tuberculosis in North Korea is to construct new governance. First, while international organizations including the UN and WHO continuously promote comprehensive tuberculosis aid projects for North Korea, they should form stronger partnerships with the governments of North Korea and South Korea, as well as private organizations. This way, they can enhance their role as mediators, enabling combination of efforts and cooperation during operation of projects. Second, the role of the North Korean government in tuberculosis control needs to be strengthened. In particular, it is necessary for the North Korean government to conduct an objective evaluation of the state of tuberculosis in North Korea at the public sphere. Further, the North Korean government must construct an effective cooperation system with the international community, as the role of the international community is especially important in this process. Third, national tuberculosis control planning and, in particular, control of MDR-TB should be promoted by international organizations, the North Korean government, and international and Korean tuberculosis specialists and their organizations. If necessary, private organizations may need to be regulated. Fourth, rather than focusing on control of MDR-TB, it is desirable for private organizations to focus on projects such as financial support, expansion of nursing facilities for tuberculosis patients in North Korea, and nutrition aid projects (e.g., support for vegetable cultivation in vinyl greenhouse, farming of frogs or mud loaches, livestock farming). However, these projects also need to be performed in close cooperation with other groups and international organizations. Lastly, the South Korean government should play a more active role in tuberculosis issues in North Korea. They need to expand their contribution to tuberculosis aid projects to North Korea, including financial support, which has been promoted by the international community from 2015. At the same time, the South Korean government should enhance their role as a mediator and specialist. From that perspective, it was highly encouraging to see the South Korean government lead a recent attempt to regularize a North Korean tuberculosis and vaccine-related meeting with international organizations and Korean non-governmental organizations. However, that is just a beginning. This meeting should become a regular meeting that includes the North Korean government and specialists.

Solutions for problems in tuberculosis aid projects in North Korea and achievement of successful outcomes require each of the following: inspection of the existing support system, maintenance of a high degree of expertise, cooperation among various related parties including the international community, mediation to induce this cooperation, a more active role of the South Korean government, and encouragement of the North Korean government to participate. Whether we can successfully control tuberculosis in North Korea will be an important touchstone to predict the future of the Korean Peninsula beyond simple tuberculosis issues.

## Figures and Tables

**Table 1.** Major TB programs of North Korea

Year	Major TB programs
1998-2003	Plan of action for the implementation of DOTS
2001-2007	GDF TB project
2007	ARTI survey
2007-2015	A multi-year strategic plan for 2008-2015 to stop TB
2010-2015	GFATM TB project
2015-2018	New strategic plan 2015-2018

TB, tuberculosis; DOTS, directly observed treatment, short course; GDF, Global Drug Facility; ARTI, annual risk of tuberculosis infection; GFATM, Global Fund to Fight AIDS, Tuberculosis and Malaria.
