# Supplementary material for: A new strategy for tuberculosis control in North Korea
Source: Epidemiol Health. 2015 Nov 22;37:e2015053. doi: 10.4178/epih/e2015053 (PMC4722223; doi:10.4178/epih/e2015053)
Supplement: Supplementary file 1 [file epih-37-e2015053-supplementary.pdf]

북한 결핵관리 무엇이 필요한가? (A new strategy for tuberculosis control of North Korea)

신영전[1], 기모란[2], 성낙문[3]

[1]한양대학교 의과대학 예방의학교실, 서울 [2]국립암센터 국제암대학원대학교 암관리정책학과, 고양

[3]마산국립결핵병원 임상연구센터, 창원

Young-jeon Shin[1], Moran Ki[2], Nackmoon Sung[3]

[1]Department of Preventive Medicine, Hanyang University College of Medicine, Seoul, Korea
[2]Department of Cancer Control and Policy, Graduate School of Cancer Science and Policy, National Cancer Center, Goyang, Korea

[3]Clincial Research Center, Masan National Tuberculosis Hospital, Changwon, Korea

Corresponding author
Young-jeon SHIN,
Department of Preventive Medicine, Hanyang University College of Medicine
222 Wangsimni-ro Sungdong-gu, Seoul, 133-791 Korea
yshin@hanyang.ac.kr
Tel 82-2-2220-0668,
Fax 82-2-2293-0660,

ORCID

Young-jeon SHIN,

Moran Ki, http://orcid.org/ 0000-0002-8892-7104

Nackmoon Sung,

초록:

결핵은 북한의 주요 질병 중 하나이다. 기존 사업이 이루어낸 일부 긍정적인 성과에도 불구하고 북한 결핵사업의 문제들을 해결하고 성공적인 성과를 거두기 위해서는 기존 지원체계에 대한 반성과 함께 고도 전문성의 지속유지, 국제사회를 포함한 다양한 관련자들간의 협력, 이러한 협력을 이끌어낼 수 있는 조정능력, 남한정부의 보다 적극적인 역할, 북한 당사자의 참여유도가 필요하다.

Key words(6 개) : Tuberculosis, Multidrug-resistant tuberculosis, North Korea, Non Governmental Organizations, International Cooperations

1. 서론

결핵은 북한의 주요 질병 중 하나이다. 특별히 결핵은 김일성의 지시에 의해 특별히 관리되는 질병이며 간염과 함께 별도의 관리, 치료 전달체계를 가지고 있다. 일반적으로 1970년대 말까지 북한은 국가차원에서 결핵을 매우 효과적으로 관리했던 것으로 알려져 있다.

결핵은 빈곤, 기근이 있는 곳에서 높은 유병률과 발생률은 보이는 질병이다. 1980년대 후반부터 시작된 북한의 경제적, 국제정치적 어려움은 1990년대 말 대기근으로 이어지고 이는 결핵관리의 실패로 이어졌다. 또한 결핵은 장기간의 정교한 치료가 필요한 질병이다. 치료기간 중 충분한 휴식과 영양이 보장되어야 한다. 전문적인 진단, 전문가에 의한 장기간의 지속적 관찰, 투약, 치료가 안 될 경우, 퇴치되지 않을 뿐만 아니라 내성결핵을 만들어 낸다. 그럼에도 불구하고 효과적인 진단, 치료, 추구관리를 위해 필요한 요소들은 1990년대 이후 북한에서 이루어내기 어려운 내용이다. 효과적인 북한 결핵관리체계 재구축을 위해 기존 사업의 문제점을 파악하고 이의 개선을 위한 전략방안을 도출하고자 한다.

2. 북한 결핵지원 사업현황

대북 결핵지원사업은 남북교류협력, 특별히 보건의료부문 사업에서 중요한 한 부분을 차지해왔다. 북한의 요구, 필요성, 상대적으로 값싼 약값 등이 교류협력을 용이하게 하는 측면이 있었다. 또한 "유진벨" 등 일부 대북협력단체처럼 결핵을 사업대상으로 삼고 활동하는 단체들이 있어 집중적인 지원이 가능하였다. 또한 1997년부터 북한 전역에 DOTS (Directly Observed Treatment, Short Course)을 진행하였고, 공식적으로는 이 작업이 매우 성공적이라고 발표하였다. 2010년부터는 글로벌펀드(Global Fund)의 대규모 재정지원을 통해 북한 전역에 포괄적인 결핵 진단, 치료, 관

리 사업이 시작되었고 최근 새로운 사업(New strategic plan, 2015-2018)을 추진할 계획이다(Table 1).

이러한 대규모 사업에도 불구하여 북한의 결핵이 통제 하에 있다는 확실한 근거는 아직 확보되지 않고 있다. 오히려 결핵치료에 반응하지 않는 환자들이 다수 발생하고 있다는 징표들이 나타나고 있다 [1].

3. 북한 결핵관리의 주요 문제점

북한 결핵관리의 가장 심각한 문제점은 신뢰할 만한 결핵관련 자료를 확보하기 어렵다는 것이다. 기존 공식자료에 대한 신뢰도에 대해서는 많은 의문이 제기되고 있다. 무엇보다 전국을 대표할만한 정기적인 서베이나 서베일런스가 이루어지지 않고 있다.

결핵의 진단영역에서는 진단, 치료결과 확인을 위한 기초적인 장비, 인력 부족으로 적절한 도말검사, 방사선 검사를 통한 진단이 이루어지지 못하고 있으며 따라서, 신뢰할 만한 국가차원 결핵현황 파악뿐만 아니라 체계적인 결핵환자 관리도 이루어지기 어려운 상황이다. 특별히, 내성결핵환자에 대한 진단능력은 극히 취약하여 적절한 관리가 어려운 상황으로 알려져 있다.

치료부문도 최근 대규모 지원사업 확대에도 불구하고, 결핵환자가 충분히 감소했다는 근거가 확인되지 않고 있다. 현재 치료부문에서 가장 큰 문제가 되고 있는 것은 도말 음성 환자문제이다. 도말 양성자에 대해서는 지속적인 관리를 하도록 하는 정책을 펴고 있으나 도말 음성환자에 대해서는 특별히 관리가 이루어지지 못하고 있다 [2]. 진단을 하더라도 식량, 주거조건 등의 미비로 장기간 요양시설에 격리가 안되고 있어 환자가 가족이나 지역사회의 감염원이 될 가능성이 매우 높다. 또한 현재 상당수의 다제내성 결핵환자가 존재하나 그 중 일부만이 치료가 이루어지고 있고, 그 치료 역시 그 적절성 여부가 충분히 확인되지 않고 있다. 심지어 최근에는 광범위내성결핵(Extensively Drug Resistant-Tuberculosis, XDR-TB) 환자의 발생도 보고되고 있다.

한편, 북한 결핵사업에 참여하고 북한당국, 국제기구, 민간단체들 간에 효과적인 역할조정이나 정보공유가 이루어지지 않고 있어 사업의 효과를 저하시키고 있다. 특별히 주요 주체별 문제점은 다음과 같다.

가. 글로벌펀드, WHO 등 국제기구 결핵사업

투입된 예산 규모에 비해 불확실한 성과

2010년 이래 4,8021,294 USD에 달하는 대규모 북한 결핵사업이 진행되었고 자체적으로 높은 평가를 받았다는 소식에도 불구하고 현재 공식적/비공식적으로 전해지고 있는 북한 결핵관련 지표만으로는 북한 결핵문제와 관리방법에 현저한 개선이 이루어졌다고 판단하기 어렵다.

무엇보다 사업의 계획, 시행, 평가에 기본이 되는 기초적인 베이스라인 결핵 서베이가 아직도 완성되지 못하고 있다. 이러한 상황에서 최근 WHO가 북한의 결핵상황을 점검하고 발간한 보고서(Report of the Joint Monitoring Mission, 2014)는 일부 결핵신고율의 향상과 같은 추가적인 정보를 제공하고 있으나 [3] 여전히 북한 결핵실태와 결핵관리사업의 효과를 평가하기에는 충분하지 못하다. 현재 진행하고 있는 베이스라인 조사가 2015년 말이나 이후에나 완료되더라도 현재까지 사업의 성과를 확인하기 충분한 정보를 가지지 못할 가능성이 높다., 또한 국가적 결핵조사 결과가 없는 상태에서 만들어진 "새로운 전략계획(New strategic plan 2015-2018)"은 기본적으로 한계를 가질 수 밖에 없다.

사업추진과정 상의 문제: '조화의 실패(Harmonization Failure)'

글로벌 펀드를 중심으로 한 대규모 북한 결핵사업의 추진은 기본적으로 바람직하지만, 그 추진은 기존 북한 결핵사업을 해 오고 있던 단체들과의 충분한 조율이 이루어지지 않은 상태에서 소위 '밀고 들어오는 방식'으로 이루어졌다. 이로 인해 기존 대북결핵 사업을 해오던 단체들은 갑작스럽게 사업을 포기하거나 전환할 수밖에 없었으며, 이들의 열의와 역량은 사용되지 못하고 버려지고 있다. 이후에도 소규모 또는 대규모 결핵약 지원이 산발적으로 이루어지고 있으나 이 역시 WHO 등 국제기구가 진행하는 대규모 사업에 통합되지 못한채 이루어지고 있다.

문제는 이렇게 통합되지 못하고 산발적으로 진행되는 결핵지원사업은 오히려 다제내성 결핵 등의 문제를 야기할 가능성이 매우 높다는 점이다. 이에 대한 지속적인 지적에도 불구하고, 이러한 '조화의 실패(harmonization failure)'는 여전히 개선될 가능성을 보이지 않고 있다.

나. NGOs와 한국정부 대북결핵사업

지원단체의 비전문성 문제

결핵지원 사업은 다른 사업과는 달리 (1) 고도의 전문성, (2) 현장성, (3) 지속적인 정교한 모니터링과 피드백, (4) 정부 행정능력과의 유기적 협력을 필요로 하는 사업이다. 이상과 같은 조건을 충분히 갖추지 못할 경우, 다제내성결핵(MDR), 광범위 약제내성 결핵(XDR)의 발생이라는 문제를 일으키고, 다제내성결핵에 대한 통제력마저 상실 할 경우, 남북한 모두 재난적 상황에 봉착할 수 있다. 이미 북한에서는 상당한 규모의 다제내성결핵 환자가 발생하고 있는 것으로 보고되고 있다. 이는 일차적으로 북한당국의 책임이지만, 그동안 결핵지원을 했던 국제기구, 국내외 민간단체들의 책임도 있다. 무엇보다 과거와 현재의 대북 결핵지원 방식들에 문제가 있었다는 것을 의미한다. 더욱이 이렇게 1차 결핵 진단, 치료, 관리에서의 문제점이 충분히 개선되지 않은 상황에서 다제내성결핵(MDR-TB) 치료를 위한 2차 결핵의 지원이 시작되고 있다. 2차 약제에 의한 치료의 적절성에 대한 정보는 전문가들과 충분히 공유되지 못하고 있다. 이는 향후 다수의 '광범위 약제내성 결

핵(XDR)' 발생 가능성을 예고하는 것이다.

지원단체 간 협력부족 문제

대북 결핵지원 사업에서 민간단체들 역시 글로벌펀드/WHO의 대규모 북한 결핵사업과 거의 연계와 역할분담을 이루지 못하고 있으며, 민간단체 간의 정보교류나 협력도 거의 이루어지지 못하고 있는 채 자기 단체의 정치력을 동원하여 결핵약 모금과 반출을 강행하는 양상이 지속되고 있다.

조직 간 역할 조정자로서의 정부역할 미비

현재 한국정부는 담당인력 부족, 결핵에 대한 전문성 부족 등으로 인해, 국제기구사업, 민간단체사업에 대한 적극적 참여, 조정을 충분히 해내지 못하고 있다.

4. 소결. 정책과제 및 제언: 약(藥)이냐 독(毒)이냐?

북한 결핵사업은 필요하지만, 제대로 돕지 않으며 호의(好意)가 '약(藥)'이 아니라 '독(毒)'이 될 수 있는 사업이다. 따라서 북한 결핵문제의 개선을 위해서는 다음과 같은 개선이 신속히 이루어져야 한다.

북한 결핵현황조사의 신속 실시와 정례화

보다 효과적인 대북결핵사업이 되기 위해서는 현재 늦어지고 있는 전국단위 결핵현황조사가 신속히 이루어져야 하고, 향후 결핵사업은 정기적으로 이루어지는 결핵현황조사에 근거하여 추진되어야 한다.

1차 예방과 치료 중심의 결핵관리사업

최근 북한결핵지원이 다제내성 결핵 지원으로 초점이 옮겨가는 것은 매우 심각한 문제이다. 가능한 빨리 지속적인 국가수준 결핵 서베이가 가동되어야 하고 결핵의 예방, 조기발견과 치료가 제대로 이루어지도록 하는 것을 우선적인 목표로 설정하여 '호미로 막을 일을 가래로 막는 우'를 범하지 말아야 한다.

다제내성 결핵문제에 대한 신중한 접근

결핵의 1차 예방과 치료가 효과적으로 이루어지고 있다는 근거가 확보되기 전에 2차 결핵약을 이용한 다제내성 치료체계를 가동시키는 것은 매우 위험한 일이다. 더욱이 다제내성결핵의 관리는 민간단체가 아니라 국제기구-북한보건성이 중심이 되어 이들간의 충분한 합의, 치료조건의 확

인 이후에 진행하여야 한다.

북한결핵관리의 ‘새로운 거버넌스’ 구축

북한 결핵과 관련한 과거, 현재의 노력과 열성에 대한 감사와 함께, 한편으로는 “과거와 지금의 방식이 최선인가?”에 대한 냉정한 평가가 필요하고 지혜를 모아야 한다. 효과적인 북한결핵관리를 위해 현 시점에서 가장 중요한 과제는 새로운 거버넌스를 구축하는 것이다. 첫째. UN, WHO 등 국제기구는 포괄적인 대북결핵사업을 지속적으로 추진하되, 북한과 한국정부, 민간단체들과 보다 강력한 파트너십을 구축하여 사업과정에서 조화와 협력이 가능하도록 조정자로서의 역할을 강화하여야 한다. 둘째, 북한결핵관리 사업에서 북한당국자의 역할을 강화할 필요가 있으며, 특별히 공론의 장에서 객관적으로 북한 결핵상태를 평가하고 국제사회와 효과적인 협력체계를 구축할 수 있도록 지원할 필요가 있다. 특별히 이 과정에서 국제사회의 역할이 중요하다. 셋째, 국가차원의 결핵관리계획의 수립과 특별히 다제내성 결핵의 관리 문제는 국제기구, 북한당국, 관련 국내외 결핵전문가와 조직이 중심이 되어 추진하는 것이 바람직하다. 넷째, 민간단체는 다제내성 결핵의 관리보다는 기금지원, 북한 결핵환자들의 요양시설 확충, 영양지원사업(예, 비닐하우스 채소재배지원, 개구리, 미꾸라지 양식, 축산 등)과 같은 사업에 초점을 맞추는 것이 바람직하다. 다만, 이 역시 단체, 국제기구와의 상호 긴밀한 협력체계 속에서 이루어져야 할 것이다. 마지막으로, 한국정부는 북한 결핵문제에 대한 보다 적극적인 역할을 수행해야 한다. 2015년부터 추진되는 국제사회 북한 결핵사업에 재정적 지원을 포함한 더 많은 기여를 하여야 한다. 이와 함께 조정자, 전문가로서의 역할을 크게 강화하여야 한다. 그런 점에서 최근 한국정부가 주도하여 북한결핵, 백신과 관련하여 국제기구, 국내 NGOs 등과의 회의를 정례화하려는 시도는 매우 바람직한 일이다. 그러나 이것은 시작에 불과하다. 이 모임을 북한 당국과 전문가를 포함하는 정기적인 모임으로 발전시켜야 한다. 북한 결핵사업의 문제들을 해결하고 성공적인 성과를 거두기 위해서는 기존 지원체계에 대한 반성과 함께 고도 전문성의 지속유지, 국제사회를 포함한 다양한 관련자들간의 협력, 이러한 협력을 이끌어낼 수 있는 조정능력 발휘, 남한정부의 보다 적극적인 역할, 북한 당사자의 참여유도가 필요하다. 우리가 북한 결핵을 성공적으로 관리할 수 있느냐 하는 것은 단지 결핵문제를 넘어 한반도의 미래를 점칠 수 있는 중요한 시금석이 될 것이다.

Acknowledgement

Conflict of interest
The author has no conflicts of interest to declare for this study.

References

1.Seung KJ, Linton SW. The growing problem of multidrug-resistant tuberculosis in North Korea. PLoS medicine 2013;10:e1001486.

2.Oh K. Current status and management of tuberculosis in North Korea. KDI Economic Review of DPRK 2015;17:23-31.

3.WHO. Report of the Joint Monitoring Mission, 2014: SEARO; 2014
